# Kinetic characterization of RNA synthesis catalyzed by the model hyperthermophilic archaeon *Thermococcus kodakarensis* RNA polymerase

**DOI:** 10.1128/msphere.00319-25

**Published:** 2025-09-26

**Authors:** Stephanie L. Cooper Horton, Thomas J. Santangelo, Aaron L. Lucius, David A. Schneider

**Affiliations:** 1 Department of Biochemistry and Molecular Genetics, Heersink School of Medicine, University of Alabama at Birmingham, Birmingham, Alabama, USA; 2 Department of Biochemistry and Molecular Biology, Colorado State University, Fort Collins, Colorado, USA; 3 Department of Chemistry, University of Alabama at Birmingham, Birmingham, Alabama, USA; Philipps University of Marburg, Marburg, Germany

**Keywords:** Archaea, RNAP, RNA synthesis, transcription, elongation complex, nucleotide addition

## Abstract

At the broadest level of taxonomy, living organisms are divided into the domains Archaea, Bacteria, and Eukarya. Despite the significant differences in cellular organization, metabolic processes, and native environments between the prokaryotic Bacteria and Archaea compared to eukaryotes, the essential biological process of RNA synthesis is generally conserved across all domains. Archaea are the progenitors of Eukarya, and the homology of the singular archaeal RNA polymerase (RNAP) and eukaryotic RNA polymerases—particularly eukaryotic RNAP II (Pol II)—highlights the common evolutionary ancestry that resulted in the modern division of transcription activities between at least three distinct eukaryotic RNAPs. While detailed kinetic evaluations of the activities of bacteria and eukaryotic RNAPs have revealed both universal and significant differences in kinetic elongation schemes, identical comparisons to archaeal-derived RNAPs have largely been absent. Here, we characterize the elongation properties of *Thermococcus kodakarensis* (*T. k*.) RNAP, a model hyperthermophilic archaeon, and compare these properties to previously characterized bacterial and eukaryotic RNAPs. We demonstrate that *T. k*. RNAP forms transcription elongation complexes even more stable than Pol II at ambient temperatures, and remarkably stable complexes at high temperatures, wherein this archaeon thrives. We surprisingly observed no significant impact of NTP concentration on the rate of nucleotide addition by the archaeal RNAP at multiple temperatures that uniquely distinguishes the archaeal RNAP from bacterial and eukaryotic RNAPs. Our results reveal how distinct regulatory strategies can be employed for the archaeal RNAP despite the overall highly conserved structure and cellular functions of multi-subunit RNAPs.

**IMPORTANCE:**

Accurate and timely regulation of gene expression is critical for survival under dynamic conditions in all living organisms. Control of transcription initiation and elongation rates is a key parameter for cellular fitness, and determination of the conserved and unique regulatory strategies that control RNA polymerase activities is of paramount importance. How RNA synthesis is catalyzed by archaeal RNA polymerases provides insight into unique and conserved regulatory strategies for survival at the limits of life.

## INTRODUCTION

 Regulated RNA synthesis is essential in all living organisms (1). In all eukaryotes, transcription of DNA to generate RNA is performed by at least three nuclear, DNA-dependent RNA polymerases (RNAPs), most typically termed Pol I, Pol II, and Pol III (1). In contrast, all in prokaryotes—which envelopes the two quite distinct domains of Bacteria and Archaea—all RNA synthesis is performed within the cytoplasm by a single DNA-dependent RNAP (2). Despite the deep evolutionary divergence between Bacteria, Archaea, and Eukarya, the overall structure and function of all multi-subunit RNAPs are well-conserved, with particular conservation of the catalytic core subunits of RNAPs (3). While the archaeal RNAP is likely ancestral to each eukaryotic RNAP, the close homology of the archaeal RNAP and eukaryotic Pol II suggests that Pol II was the primordial eukaryotic RNAP that gave rise to Pol I and Pol III (and Pol IV and Pol V in plants (2, 3).

 We previously characterized and contrasted the enzymatic properties of eukaryotic Pols I, II, and III, as well as mycobacterial RNAPs (4–10). To further our understanding of RNA synthesis in organisms across the domains of life, we examined the enzymatic properties of *Thermococcus kodakarensis* (*T. k*.) RNAP. *T. kodakarensis* is a marine archaeon isolated from shallow solfataras and hydrothermal vents (11, 12). Despite the extreme conditions in which *T. kodakarensis* is naturally found, it has been well-adapted to anaerobic laboratory growth at 85°C, making it a model hyperthermophilic archaeal species (13, 14).

 Here, we present a biochemical characterization of *T. k*. RNAP under conditions wherein the kinetic properties of the archaeal-derived RNAP can be most directly compared to bacterial and eukaryotic RNAPs. Using *in vitro* transcription techniques that allow rapid kinetic measurements, we identify enzymatic properties unique to this hyperthermophilic archaeal RNAP that collectively elucidate some of the intricacies of archaeal RNA synthesis and provide insight into enzymatic properties unique from eukaryotic and bacterial RNAPs that may be critical for survival in extreme conditions.

## RESULTS

 The structure of *T. k*. RNAP was resolved to reveal significant homology to eukaryotic Pol II (14). We hypothesized that *T. k*. RNAP activity would demonstrate temperature sensitivity, considering the species’ natural environment, but would catalyze a reaction mechanism similar to nucleotide addition catalyzed by Pol II. To elucidate the nucleotide addition cycle of *T.k*. RNAP, as well as to compare the elongation characteristics of bacterial, archaeal, and eukaryotic RNAPs under identical conditions, we performed *in vitro* transcription experiments to characterize the biochemical properties of *T. k*. RNAP.

**Fig 1:**
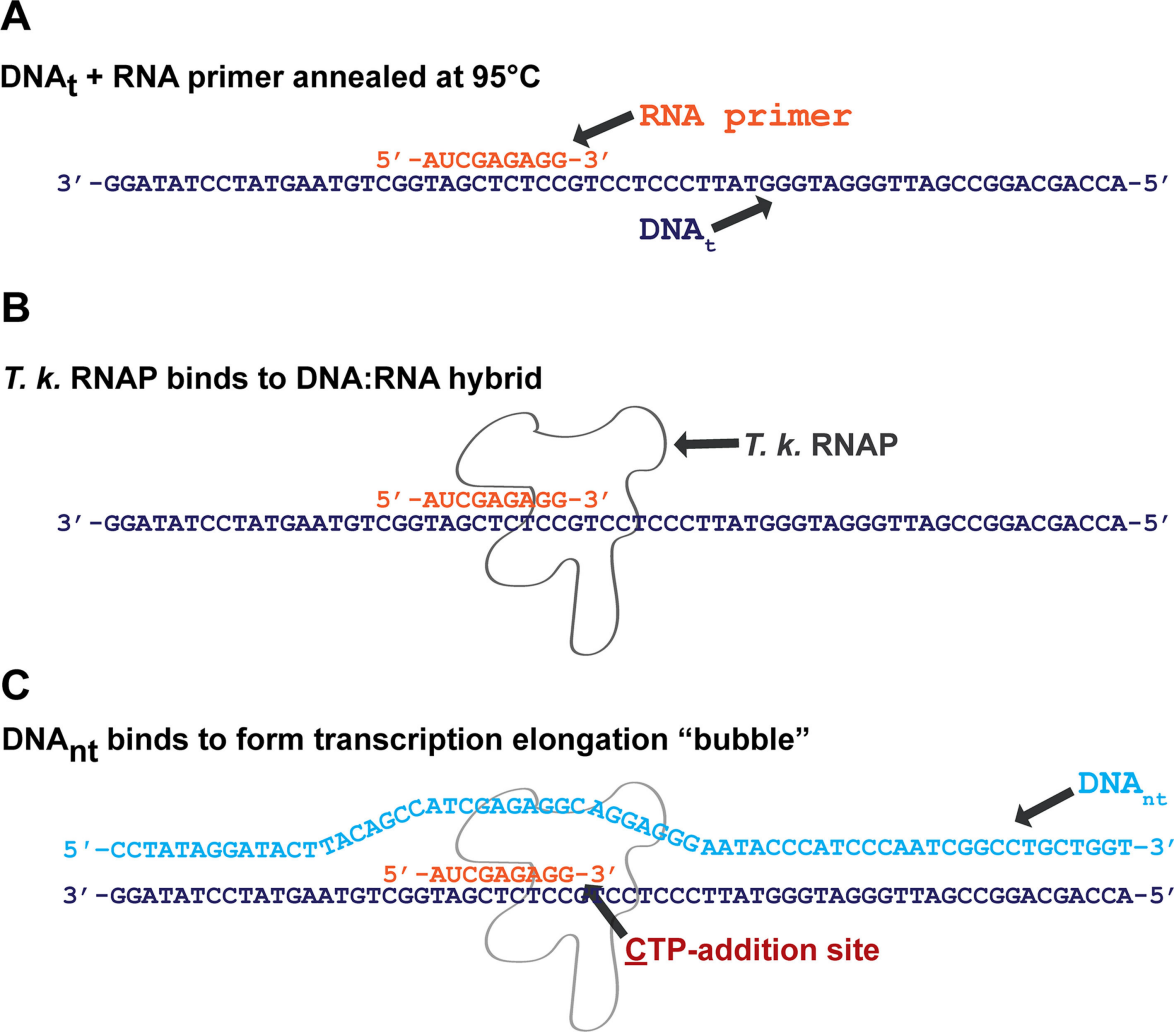
Graphical representation of promoter-independent *in vitro* transcription scaffold formation steps. (**A**) The purified DNA template (DNA_t_) strand of a total 64 nt in length is annealed to the purified 9-nt RNA primer at 95°C. Due to sequence complementarity, the RNA primer anneals to (5′−3′) nucleotide positions 36–44 of DNA_t_. (**B**) The purified T. k. RNAP is provided to the DNA:RNA hybrid, which binds by affinity to the short hybridized oligonucleotides. (**C**) The purified DNA non-template (DNA_nt_) complementary strand is provided to the RNAP-bound DNA:RNA hybrid, which binds to the DNA_t_ by sequence complementarity upstream and downstream of the transcription “bubble” occupied by the RNAP.

Given our aim of direct comparisons of the nucleotide addition cycle and elongation kinetics between RNAPs from the three domains, but the radically different DNA sequence, co-factor, energy, and basal transcription factor requirements for transcription initiation strategies employed by Bacteria, Archaea, and Eukarya, we assembled archaeal transcription complexes on a synthetic nucleic acid scaffold that supports promoter-independent formation of transcription elongation complexes (ECs; Fig. 1). This approach is generally described as scaffold-dependent transcription initiation, and it relies on innate affinity of RNA polymerase for short RNA:DNA hybrids. After the enzyme associates with the hybrid, the complex is incubated with a fully complementary non-template DNA strand, resulting in a double-stranded DNA template with a fully formed elongation complex with a short nascent RNA. This complex provides an ideal platform for comparison of diverse RNAP activities. Using the same DNA/RNA sequences for bacterial, eukaryotic, and archaeal RNA polymerases helps ensure that any differences observed in their activities are due to the innate properties of the polymerases themselves—not differences in the templates used in the reactions.

### *T. k*. RNAP transcription ECs are stable over the course of days at both high and low temperatures

**Fig 2:**
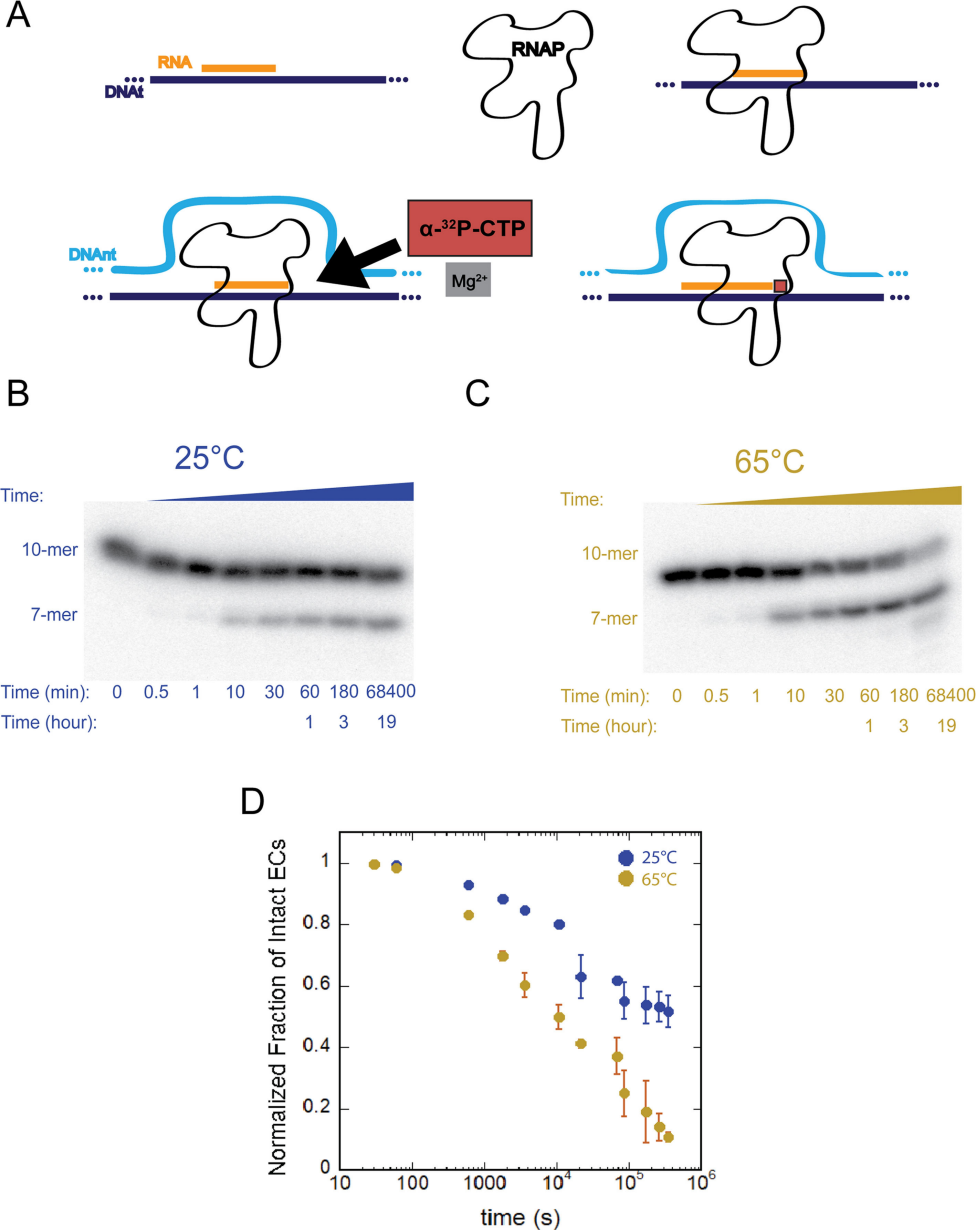
*T. k*. RNAP ECs are exceptionally stable at low and high temperatures. (**A**) Schematic of elongation complex formation and radiolabeling. DNAt = template DNA, DNAnt = non-template DNA. (**B**) and (**C**) Representative gel images of RNase protection experiments at 25°C (**B**) and 65°C (**C**). (**D**) Plot of time courses performed at both temperatures showing a normalized fraction of intact ECs over the course of 96 hours. Data points represent the average of three experimental replicates, and error bars represent the calculated standard deviation.

 For our characterization of *T. k*. RNAP, we selected two temperatures to perform a series of *in vitro* experiments: 25°C as ambient temperature (to allow direct comparisons to the biochemical and kinetic properties of bacterial and eukaryotic RNAPs) and 65°C, which is near the upper limit of the instruments used for transcription assays and within the range of temperatures where this species thrives (~60°C–98°C) (12). We first assessed the overall stability of transcription elongation complexes (ECs) formed by *T. k*. RNAP with a synthetic nucleic acid scaffold used for *in vitro* transcription analyses (Fig. 2A). Following EC assembly (EC_+9_), α-^32^P-CTP is provided and incorporated as the next cognate nucleotide forming the 10-mer RNA elongation complex (EC_+10_) while radiolabeling the nascent RNA to both provide a quantifiable signal for analysis and ensure that all of the subsequent kinetic data is derived from catalytically active ECs. The incorporated radiolabeled CMP is designated by underlining, presented as C in the sequence. As the next cognate substrate (ATP) is omitted, transcription elongation beyond 10 nts is prevented; subsequent addition of EDTA chelates Mg^2+^, which further eliminates the NTP incorporation.

 Catalytically active EC_+10_ is subjected to a destabilizing salt solution and RNase A to monitor the kinetics of disassembly of EC_+10_. While intact EC_+10_ fully encapsulates and thus protects the nascent RNA from RNase A cleavage, dissociation of the RNAP releases the 10 nt RNA (5′-AUCGAGAGGC-3′) into solution and permits RNaseA-mediated cleavage downstream of the only internal C in the 10-mer RNA, thus removing the three 5′ most nts and converting the 3′-radiolabeled 10-mer RNA to a 3′-radiolabeled 7-mer RNA. Quantification of the relative signal intensities of radiolabeled 10-nt and 7-nt RNA species over time in the destabilizing salt solutions defines the proportion of intact EC_+10_ (Fig. 2B through D).

 Previous characterization of eukaryotic Pols I, II, and III revealed a two-order of magnitude difference in eukaryotic EC complex stability: Pol II ECs are the most stable of the eukaryotic Pols, with complexes collapsing over the course of ~72 hours at 25°C (4), whereas Pol III ECs remain intact on the order of ~3 hours (5), and ECs formed with Pol I are the least stable, collapsing within just ~40 minutes (4, 5). EC_+10_ stability assays formed under identical conditions with T.k. RNAP revealed exceptionally long-lived EC_+10_ irrespective of temperature. Interestingly, we found that under the same experimental conditions and temperature *T. k*. EC_+10_ is stable >96 hours (Fig. 2B and D). Even at the elevated temperature of 65°C, *T. k*. ECs remained intact longer than 24 hours (Fig. 2C and D). Our results demonstrate that *T. k*. RNAP provides substantial stabilization of the RNA:DNA hybrid as the experimental higher temperature of 65˚C well exceeds the predicted melting point of the RNA:DNA hybrid (~30°C).

### Single-nucleotide addition catalyzed by *T. k*. RNAP is temperature-, but not NTP-concentration dependent

**Fig 3:**
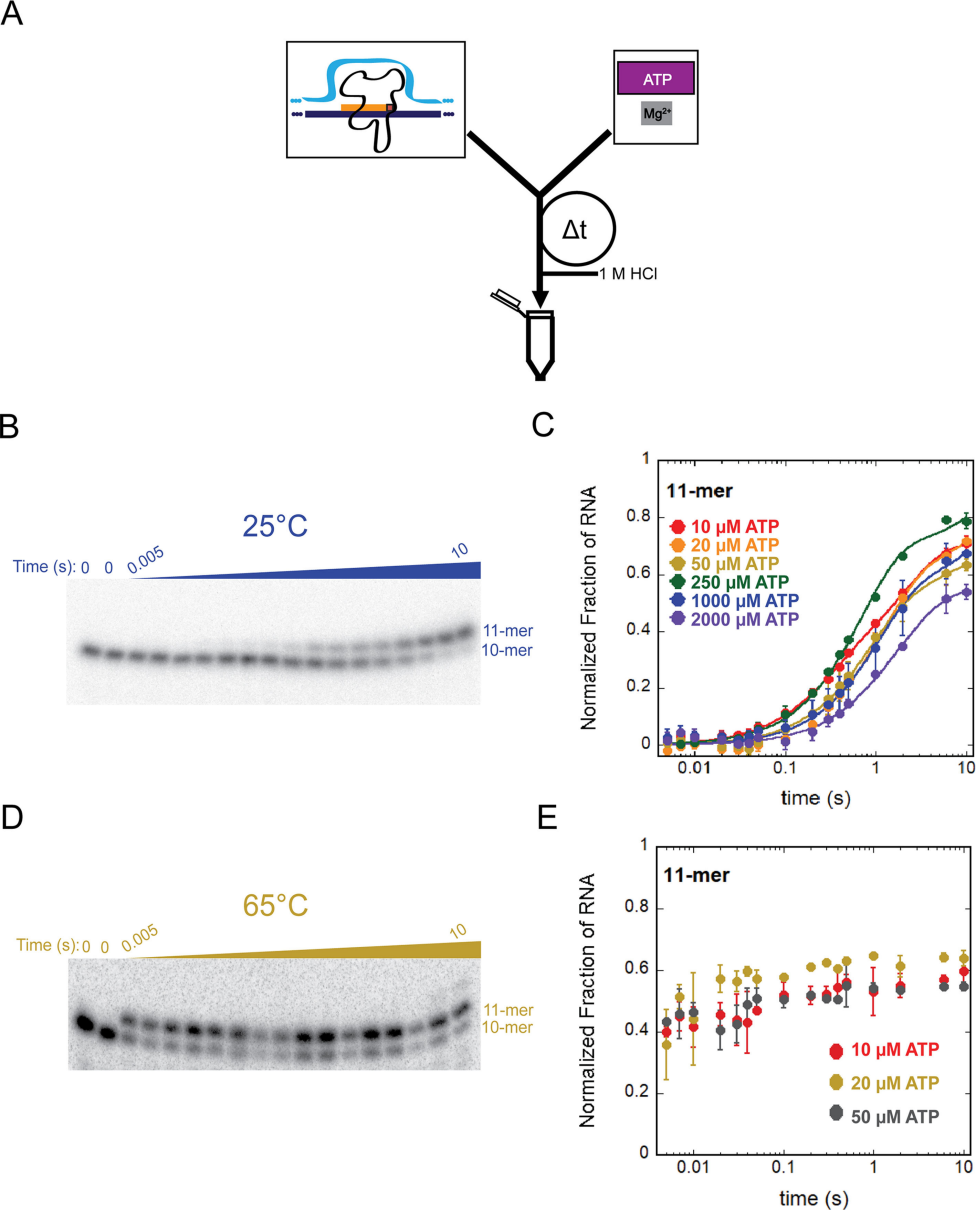
The rate of transcription elongation by *T. k*. RNAP is independent of (ATP) at ambient and high temperatures, and elongation rates are dramatically increased at near-physiological temperatures. (**A**) Schematic of the chemical quench flow experimental design. (**B**) Representative gel image of a single nucleotide addition performed at 25°C. (**C**) Plot of (ATP) titration data points collected at 25°C fit using equation 2. Data points represent the average of at least three experimental replicates, and error bars represent the calculated standard deviation. (**D**) Representative gel image of single-nucleotide addition performed at 65°C. (**E**) Plot of (ATP) data points collected at 65°C. Data points represent the average of at least three experimental replicates, and error bars represent the calculated standard deviation.

 To characterize the mechanism of *T. k*. RNAP-catalyzed nucleotide addition, we used our established promoter-independent, rapid-mixing, *in vitro* transcription experiment (4–10) to monitor nucleotide addition upon the addition of single or subsets of NTPs at different concentrations on the ms timescale. Radiolabeled EC_+10_ is loaded into a chemical quench-flow instrument opposite a solution of ATP, the cofactor magnesium, and heparin (detailed in Materials and Methods). ATP serves as the substrate for nucleotide addition to convert EC_+10_ to EC_+11_, and heparin acts as a trap for unbound RNAPs, ensuring single-turnover reaction conditions (4–7, 15). The contents of the two syringes are mixed within 2 ms, and the nucleotide addition reaction is allowed to proceed for a user-determined period of time—ranging from 5 ms to 10 s—before being rapidly quenched with hydrochloric acid (Fig. 3A). RNA products from each time point are then separated via denaturing polyacrylamide gel electrophoresis that provides resolution of radiolabeled 10 nt and 11 nt RNA. Density of each radiolabeled RNA product is measured by phosphorimage analysis and model-independent analysis using KaleidaGraph (Fig. 3B through E) (4–7).

 Time courses describing a single-nucleotide addition event catalyzed by *T. k*. RNAP were collected both at 25°C and 65°C, over a range of ATP concentrations (Fig. 3B and D). We observed negligible ATP concentration dependence for the formation of the 11-mer over time at 25°C (Fig. 3C). We note that 250 µM ATP demonstrated a higher amplitude of signal. The amplitude does not correlate with the rate constant governing the nucleotide addition but rather reflects variation in signal to noise, due to the degree of decay or “freshness” of ^32^P-radionucleotides. Therefore, no obvious trend is apparent in the titration time courses. The fact that nucleotide addition rate is independent of ATP concentration highlights a unique feature of *T.k*. RNAP (and perhaps other archaeal RNAPs), which contrasts with both eukaryotic and bacterial RNAPs that all demonstrate clear ATP-concentration dependence for nucleotide addition (4–10).

To extract values for the observed rate constants (k_obs_) governing nucleotide addition, the data were fit using nonlinear least squares (NLLS) to a sum of two exponentials (equation 1), since the data could not be described well by fitting to a single exponential (equation 2; Fig. S1).


(1) $$RNA\;fraction=A_{k_{fast}}(1-e^{-(k_{fast})t})+A_{k_{slow}}(1-e^{-(k_{slow})t})$$

(2) $$RNA\;fraction=A(1-e^{-(k_{obs})t})$$

At 25°C, nucleotide addition by *T.k*. RNAP—at nearly all ATP concentrations—was best fit to a sum of two exponentials (equation 1). Fitting to a sum of two exponentials results in two observed rate constant values of k_obs_,_fast_ = (1.47 ± 1.14) s^−1^ and k_obs,slow_ = (0.35 ± 0.58) s^−1^. These rate constant values are one to two orders of magnitude slower than nucleotide addition rates (~9–67 s^−1^) catalyzed by eukaryotic Pols I, II, and III and bacterial RNAPs at the same temperature, same conditions, and from identical scaffolds (4–7).

### Model-dependent analysis of single-nucleotide addition catalyzed by *T. k*. RNAP at 25°C

Following model-independent analysis of time courses collected at 25°C, we determined the simplest kinetic model, or scheme, to describe a single-nucleotide addition event by *T.k* RNAP. Using the MATLAB tool MENOTR, we globally fit the time course data to Scheme 1.


$$EC_{10}+ATP\overset{k_{1}}{\underset{k_{d}}{⇌}}EC_{10}\cdot ATP\overset{K3}{\to }EC_{11}+PPi$$

**Fig 4:**
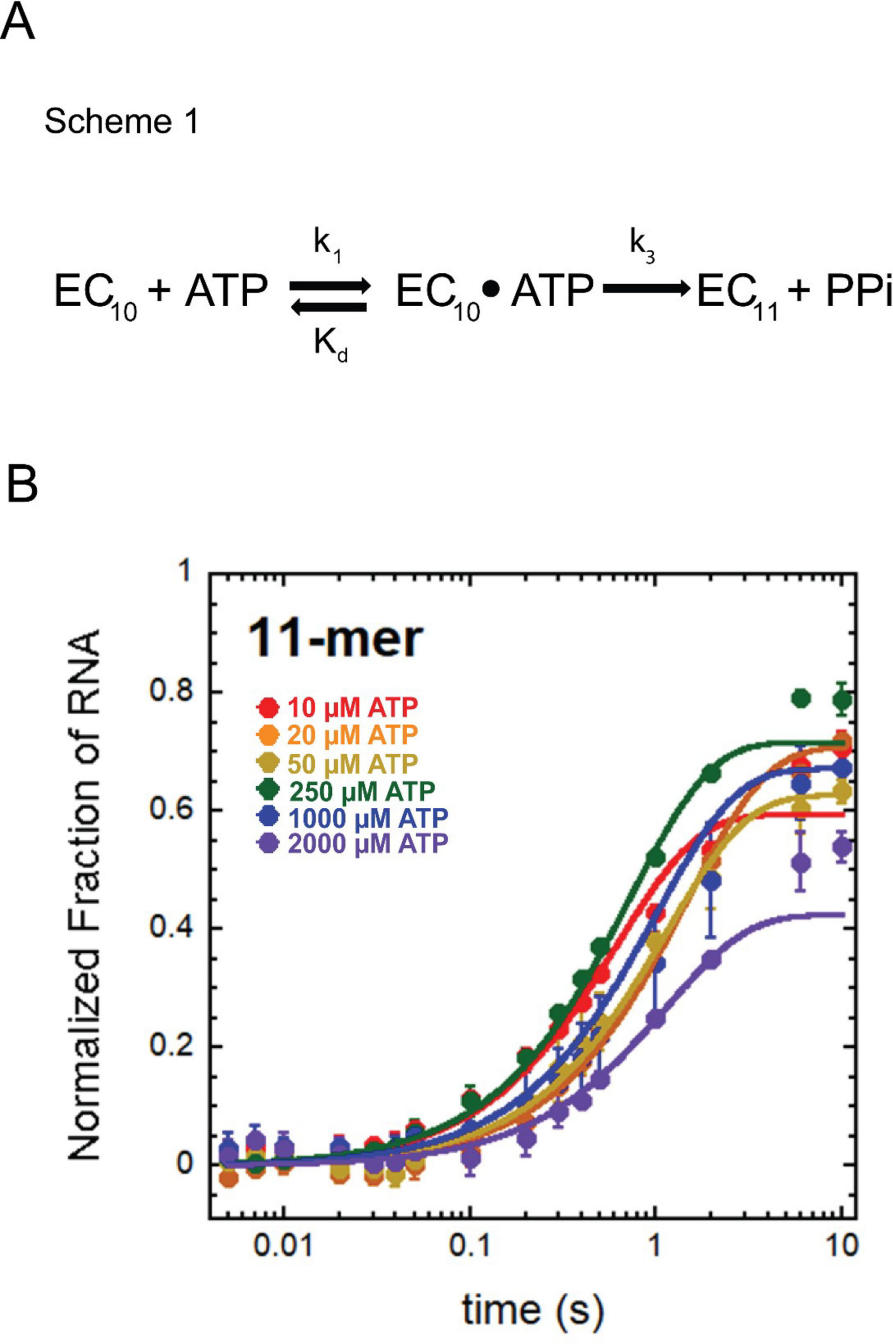
Model-dependent analysis of single-nucleotide addition at 25°C. (**A**) Scheme 1 was used in the MENOTR analysis of single-nucleotide addition time courses performed at 25°C. This scheme describes a reversible (ATP)-binding step followed by an irreversible bond formation step with elementary rate constant k_3_ resulting in formation of the 11-mer RNA. Pyrophosphate (PPi) release is undetectably fast in this experimental design and was therefore not quantified but is assumed to be coupled to the final step of the scheme. (**B**) Global model-dependent fitting of single-nucleotide addition time courses performed at 25°C. Data points represent the average of at least three experimental replicates, and error bars represent the calculated standard deviation. Parameter values are presented in Table 1, X^2^ value represents the reliability of the fit.

Scheme 1 describes ATP binding to the EC_+10_, governed by the elementary rate constant k_1_, which is fixed to the diffusion limit of 1 × 10^8^ M^−1^ s^−1^ (Fig. 4A) (15, 16). The binding step is reversible, as described by K_d_, representing the measure of binding affinity. Bond formation and extension from EC_+10_ to EC_+11_ are governed by k_3_. This scheme describes all detectable, rate-limiting steps. Our technique has millisecond temporal resolution but is not sensitive to potentially faster steps in the nucleotide addition cycle (6, 7).

Global fitting the nucleotide addition at all ATP concentrations at 25°C revealed a bond formation step with the elementary rate constant k_3_ ~1.00 s^−1^ (upper and lower bounds listed in Table 1). This rate constant is in agreement with model-independent calculations but is much slower than bacterial and eukaryotic RNAPs at 25°C (~9–67 s^−1^) evaluated on the same nucleic acid scaffold (4–7). In addition to bond formation, the substrate binding affinity (K_d_) was calculated to be ~3 µM (upper and lower bounds listed in Table 1). Again, this value is significantly lower than for other RNAPs investigated. The simplest interpretation of these data is that at 25°C, *T. k*. RNAP binds the nucleotide so tightly that bond formation is disfavored (4–8). Both the slow rate constant of bond formation and the high substrate binding affinity may be due to temperature-dependent changes in the active center of *T.k*. RNAP, which are relieved at higher temperatures.

**TABLE 1:** Global model-dependent fitting of single-nucleotide addition at 25°C^*a*^

Parameter	s^−1^	Lower bound	Upper bound
k_1_	1 × 10^8^	–^*b*^	–
K_d_	2.985 µM	2.976 µM	2.996 µM
k_3_	1.001	0.998	1.003
X^2^	15.7		

^*a*^ Lower and upper bounds were determined via grid search analysis, which represent a 68% confidence interval. X^2^ values represent the reliability of the fit. Kd represents the measure of binding affinity. The elementary rate constant k3 represents the bond formation step.

^*b*^ "–" indicates no upper or lower bound, since the parameter was fixed, not floated.

We hypothesized that at higher temperatures similar to the native environment in which *T. k*. thrives, the rate of nucleotide addition would be more similar to rates established for bacterial and eukaryotic RNAPs. While only the archaeal enzyme could be monitored for catalysis at higher temperatures, comparisons of single-nucleotide addition reactions at both 25°C and 65°C provided insightful information into the temperature dependence of *T.k*. RNAP for transcription elongation. While transcription elongation rates at 25°C were one to two orders of magnitude slower than bacterial and eukaryotic RNAPs, when identical reactions were performed at 65°C, conversion of EC_+10_ to EC_+11_ was completed prior to the first time point (5 ms) at all ATP concentrations (Fig. 4D and E). Because EC_+11_ accumulation is complete before the earliest attainable time point, we can only estimate a lower limit for the observed rate constants for a single-nucleotide addition at 65°C. Fit to a single exponential with equation 2, we determined the lower limit to be 301 ± 65 s^−1^. Notably, this rate constant is more than two orders of magnitude faster than the more robust observed rate constant at 25°C (just 1.47 ± 1.14 s^−1^), and now faster—and perhaps much faster—than bacterial and eukaryotic RNAPs. As was observed at 25˚C, nucleotide addition by *T.k*. RNAP at 65˚C did not display ATP-concentration dependence.

### Model-dependent analysis of multiple nucleotide addition events catalyzed by *T. k*. RNAP

Given that *T.k*. RNAP incorporates a single nucleotide at 65˚C faster than we can measure, even with rapid mixing, we turned to measuring multiple consecutive nucleotide addition events to more accurately establish the kinetics of nucleotide addition by this archaeal RNAP at different temperatures and NTP concentrations. To observe quantifiable nucleotide addition at both 25°C and 65°C, we employed the same promoter-independent *in vitro* transcription procedures previously described with a single alteration: the addition of GTP as well as ATP in solution. Based on the template DNA sequence, incubation with ATP and GTP will result in nine successive nucleotide addition events, extending the 10-mer nascent RNA to a 19-mer with the sequence 5′-AUCGAGAGGCAGGAGGGAA-3′. The cognate nucleotide at position +20 is UTP, which is not provided in solution.

 Similar to single-nucleotide addition time courses with *T. k*. RNAP, we observed much faster multi-nucleotide addition at 65°C compared to 25°C. Time courses of four concentrations of ATP and GTP were collected at 25°C (Fig. S2). Of the nine potential RNA intermediates, at 25°C, only three RNA species were observed over our 10 s time course: the 11-mer, 12-mer, and 13-mer (Fig. S2). This observation suggests that not only is the rate constant governing nucleotide addition slow, but transcription elongation beyond a few nucleotides by the *T.k*. RNAP is inhibited, implying deficiencies in translocation at 25˚C.

 The 25˚C time courses were subjected to global minimization using MENOTR and Scheme A (Fig. S3), which includes a single rate constant between each product. To account for trace amounts of extension products of 13-mer and above, we modeled the formation and decay of the 11-mer, 12-mer, and the formation of the 13-mer + RNA species (defined as all RNA species at least 13 nucleotides long). We assessed the reliability of fit using an irreversible scheme but found that the data points were not well described (Fig. S3).

**Fig 5:**
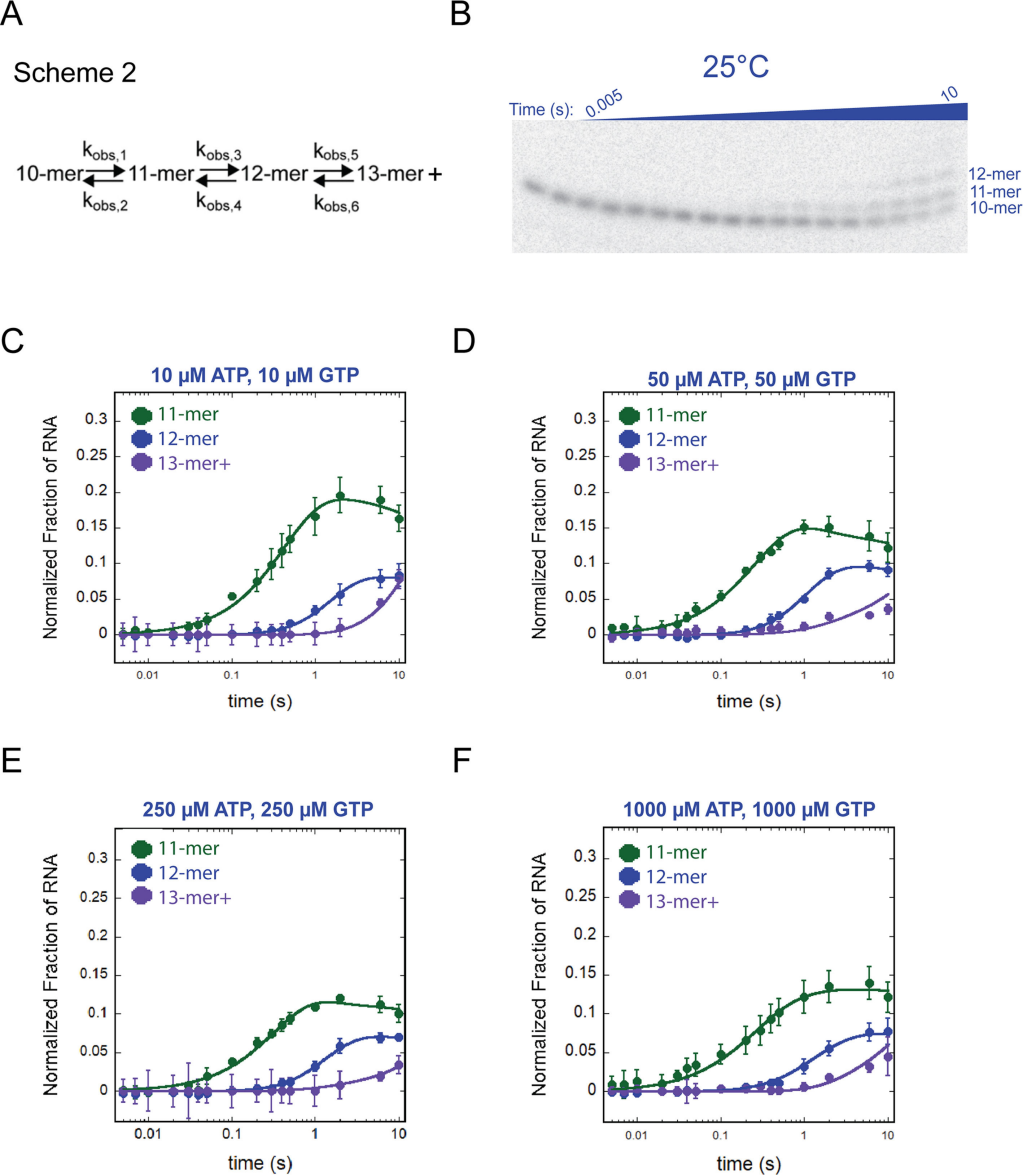
Multiple nucleotide addition events catalyzed by *T. k*. RNAP at 25°C. (**A**) Scheme 2 was used in the MENOTR model-dependent analysis of 25°C time courses. This scheme describes reversible observed rate constants between each intermediate RNA and the final 13-mer + RNA products. The test of an irreversible scheme is present in Fig. S2. (**B**) Representative gel image of multi-nucleotide addition performed at 25°C. (**C–F**) Plots of multiple nucleotide addition events performed at 25°C. Each plot is labeled at the top with the (ATP, GTP). Data points represent the average of at least three experimental replicates, and error bars represent the calculated standard deviation.

Next, we modified Scheme A to include reversibility between each RNA intermediate formation, described by the chemical reaction Scheme 2 (Fig. 5A). The requirement for reversibility between RNA intermediates has been previously observed for other RNAPs and suggests that pyrophosphate may be transiently sequestered in the active site after phosphodiester bond formation (6, 10). Visualization of a representative time course is presented in Fig. 5B. The corresponding observed rate constant values for each ATP & GTP concentration are listed in Table 2. We again observe very slow rate constants and no significant NTP-concentration dependence of nucleotide addition, which agrees with the single-nucleotide addition results at 25°C (Fig. 5C through F; Table 2).

**TABLE 2:** Model-dependent analysis of multiple nucleotide addition events at 25°C^*a*^

Parameter (s^−1^)	10 µM ATP, 10 µM GTP	50 µM ATP, 50 µM GTP	250 µM ATP, 250 µM GTP	1,000 µM ATP, 1,000 µM GTP
k_obs,1_	0.786 ± 0.25	1.351 ± 0.54	1.448 ± 0.57	0.558 ± 0.14
k_obs,2_	1.174 ± 0.39	2.656 ± 0.67	0.733 ± 0.18	2.674 ± 0.17
k_obs,3_	0.356 ± 0.01	0.772 ± 0.12	0.585 ± 0.13	0.683 ± 0.19
k_obs,4_	0.715 ± 0.06	1.064 ± 0.34	0.863 ± 0.26	1.037 ± 0.29
**k_obs,5_**	**0.145 ± 0.08**	**0.167 ± 0.02**	**0.063 ± 0.02**	**0.299 ± 0.14**
k_obs,6_	0.262 ± 0.12	0.337 ± 0.34	0.019 ± 0.02	0.429 ± 0.40
X^2^	0.00128	0.00200	0.00131	0.00415

^*a*^ Average and standard deviation were calculated for each observed rate constant (kobs) captured at 25°C as presented in Scheme 2. X^2^ values represent the reliability of the fit. The elementary rate constant k5 is highlighted in bold to represent the bond formation step.

**Fig 6:**
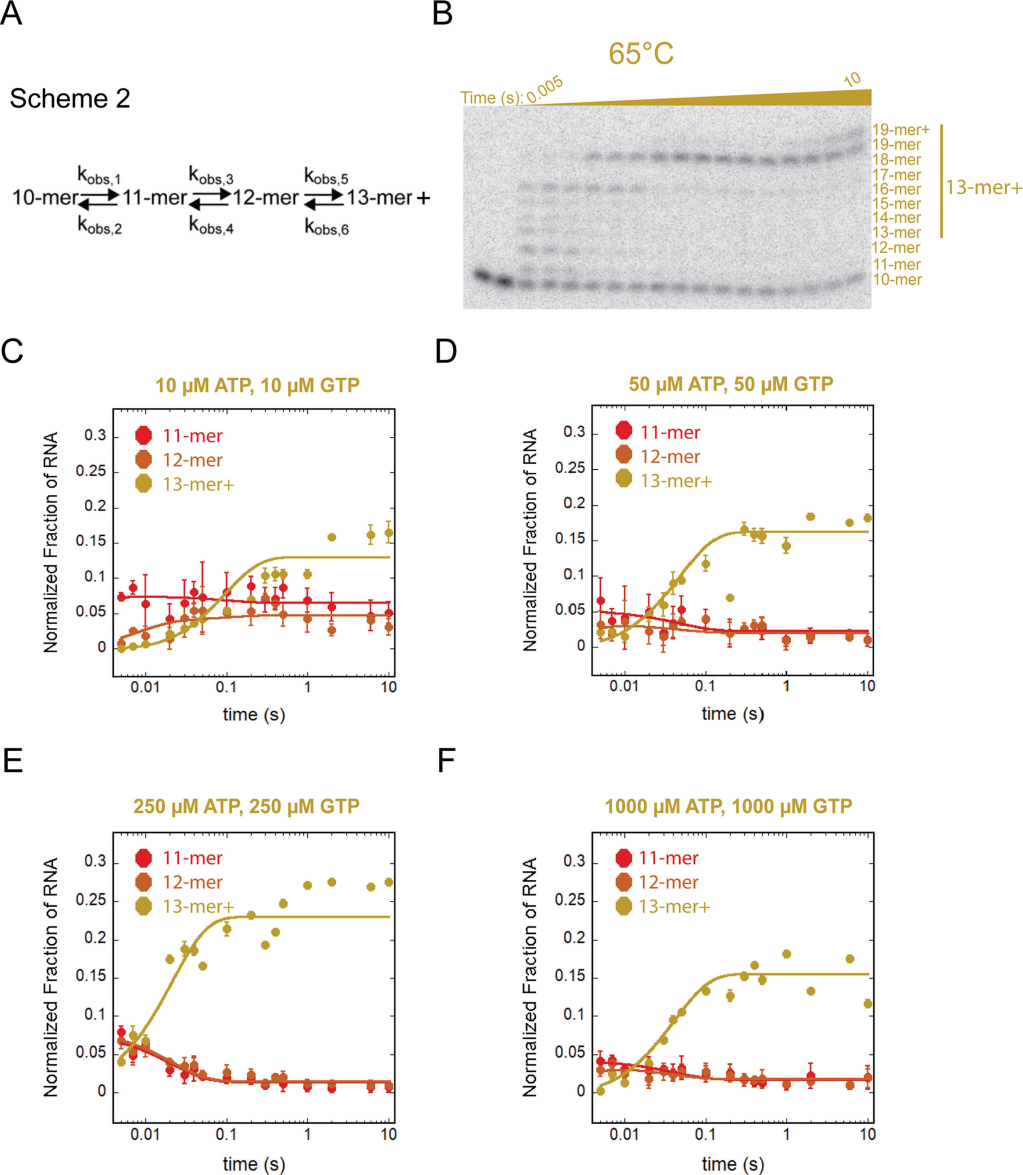
Multiple nucleotide addition events catalyzed by *T. k*. RNAP at 65°C. (**A**) Scheme 2 was used in the MENOTR model-dependent analysis of 65°C time courses. This scheme describes reversible observed rate constants between each intermediate RNA and the final 13-mer + RNA products. (**B**) Representative gel image of multi-nucleotide addition performed at 65°C. (**C–F**) Plots of multiple nucleotide addition events performed at 65°C. Each plot is labeled at the top with the (ATP, GTP). Data points represent the average of at least three experimental replicates, and error bars represent the calculated standard deviation.

We also performed multi-nucleotide addition experiments with the same ATP and GTP concentrations at 65°C. We utilized the same reaction scheme to describe the formation and decay of the 11-mer, 12-mer, and formation of the 13-mer + species (Fig. 6A). At 65°C, nucleotide addition is much faster (Fig. 6B). We again observed that at the earliest attainable time point for our equipment, formation of the 11-mer had already occurred. Similarly, the 12-mer species was also present at the earliest time point for all ATP and GTP concentrations tested (Fig. 6C through F). To more confidently determine the rate of RNA synthesis over time for *T. k*. RNAP at 65°C, we evaluated the formation of the 13-mer + species and used the formation of these RNA products to determine the average observed rate constant describing nucleotide addition. Plots of multi-nucleotide addition at 65°C are presented in Fig. 5, with corresponding observed rate constants in Table 3. To ensure that this method of analysis did not exclude usable data in the formation of the 11-mer and 12-mer RNAs, we determined the half-life of these species at each NTP concentration. Half-life values were determined to be just 3.4 ± 0.8 ms, which remains faster than the detectable limit (5 ms) of the chemical quenched-flow, meaning these RNA intermediates cannot be quantified.

**TABLE 3:** Model-dependent analysis of multiple nucleotide addition events at 65°C^*a*^

Parameter (s^−1^)	10 µM ATP, 10 µM GTP	50 µM ATP, 50 µM GTP	250 µM ATP, 250 µM GTP	1,000 µM ATP, 1,000 µM GTP
k_obs,1_	62.95 ± 6.86	2185.5 ± 563	5910.8 ± 190	373.1 ± 46.6
k_obs,2_	793.9 ± 6.16	8484.7 ± 21.6	6069.3 ± 293	1029.8 ± 228
k_obs,3_	72.98 ± 11	287.7 ± 61.8	4614.4 ± 146	384.2 ± 4.14
k_obs,4_	109.7 ± 28.2	313.2 ± 57.3	54.51 ± 54.33	382.6 ± 12.4
**k_obs,5_**	**24.2 ± 10.5**	**141.3 ± 89.2**	**135.2 ± 1.97**	**107.8 ± 10**
k_obs,6_	9.05 ± 3.68	17.7 ± 4.76	7.65 ± 0.75	13.96 ± 1.97
X^2^	0.01659	0.03424	0.00981	0.01062

^*a*^ Average and standard deviation calculated for each observed rate constant (kobs) captured at 65°C as presented in Scheme 2. X^2^ values represent the reliability of the fit. The elementary rate constant k5 is highlighted in bold to represent the bond formation step.

**Fig 7:**
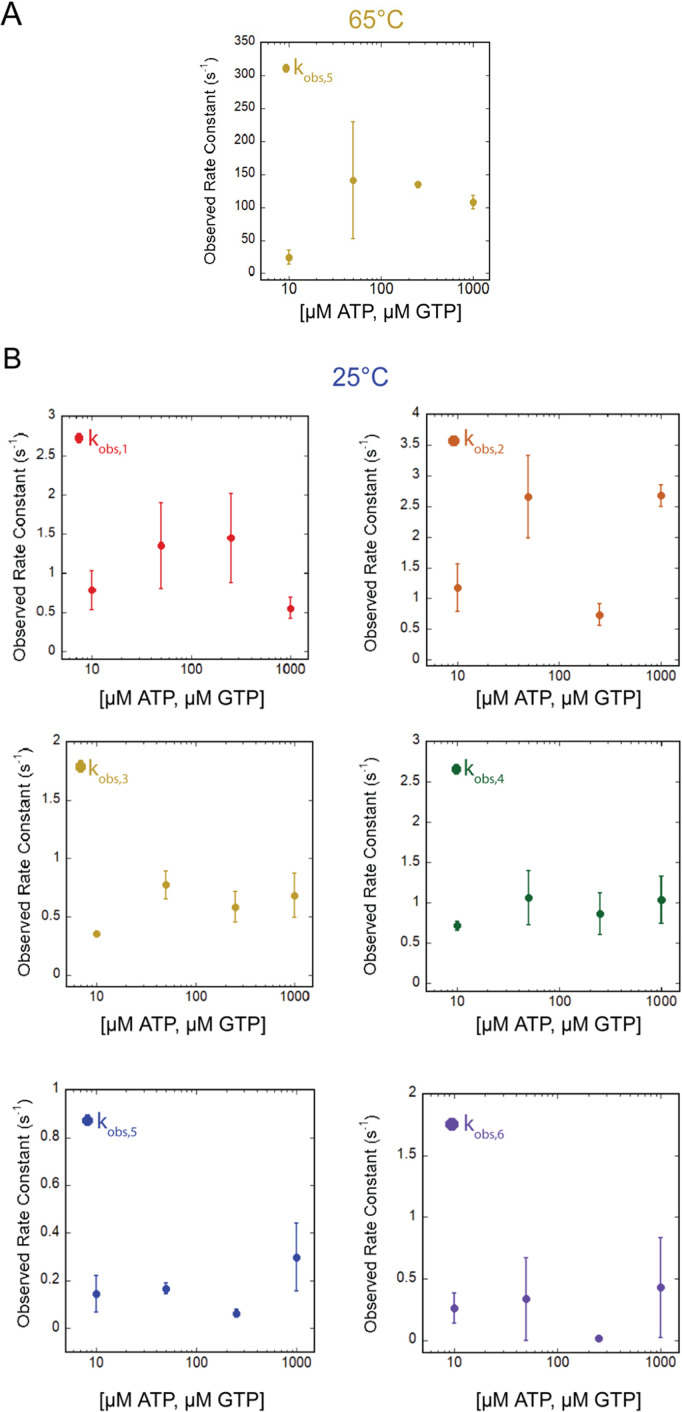
Rate-limiting steps of elongation are independent of (NTP). (**A**) Secondary plot of the representative observed rate constant k_obs,5_ for time courses performed at 65°C. (**B**) Secondary plots of all observed rate constants determined for time courses performed at 25°C. For all plots, data points represent the average observed rate constant from at least three experimental replicates, and error bars represent the calculated standard deviation.

We highlight k_obs,5_ as descriptive of nucleotide addition catalyzed by *T. k*. RNAP at 65°C with an average of 102 ± 46.7 s^−1^ across each of four NTP concentrations (Fig. 7A; Table 3). This value is once again two orders of magnitude greater than the rate constant measured under identical conditions at 25°C. In the kinetic mechanism used to describe nucleotide addition, k_obs,5_ describes bond formation and extension of the RNA to higher-order oligomers. At NTP concentrations > 10 µM, we do not observe significant NTP-concentration dependence on the rate constant describing bond formation. It is plausible that 10 µM NTP concentration is a low enough concentration to challenge the assumptions of rapid equilibrium, as we have observed for other RNAPs at 25°C (4–7), or that RNAPs have evolved to respond to NTP deprivation as a stress response (17).

For *T. k*. RNAP nucleotide addition at 25°C, we do not observe significant NTP-concentration dependence on any kinetic constant or parameter value (Fig. 7B; Table 2). The lack of significant NTP-concentration dependence on the rate constant describing bond formation has not been observed for eukaryotic or bacterial RNAPs characterized on identical synthetic nucleic acid scaffolds (4–7). The lack of NTP-concentration dependence in the rate constants governing nucleotide addition by *T. k*. RNAP suggests that nucleotide binding may be decoupled from bond formation at 25°C. Due to the very slow rate constant describing bond formation, we hypothesize that at 25°C, the rate-limiting factor is not NTP abundance, but rather slow isomerization of an enzyme conformation to permit nucleotide addition. At 65˚C, nucleotide addition rates are two orders of magnitude faster, but once again display no obvious NTP-concentration dependence, suggesting that temperature influences overall nucleotide addition kinetics, but that NTP concentration is not limiting for *T.k*. RNAP.

## DISCUSSION

In this study, we characterized the enzymatic properties of *T. k*. RNAP using a series of *in vitro* transcription experiments to elucidate the unique enzymatic properties of this model hyperthermophilic archaeal RNAP and draw comparisons to bacterial and eukaryotic RNAPs under near identical conditions. Three unexpectedly unique features of the T.k. enzyme emerged from this study: (i) *T. k*. RNAP forms an exceptionally stable transcription elongation complex, (ii) *T. k*. RNAP is highly sensitive to the reaction temperature, and (iii) *T. k*. RNAP is not responsive to changes in NTP concentration in the physiological range.

*T.k*. RNAP forms remarkably stable ECs (Fig. 2) that are longer lived than even Pol II ECs under the same experimental conditions (4). The longevity of *T. k*. ECs suggests that environmental pressures of hyperthermophilic growth have resulted in ECs stable enough to remain intact at temperatures that would likely compromise and potentially denature RNAPs from mesophilic organisms (18)(insert reference former #23 here). Furthermore, unlike bacterial RNAP, many archaeal enzymes must navigate histone-based chromatinized templates (19)(20)(21)(22)(insert references former #22, #24, #25, and #28 here). Perhaps this barrier in the context of limited chromatin modifiers selected for highly stable transcription elongation complexes. Given the protein thermo-instability of mesophilic eukaryotic and most bacterial RNAPs, direct comparison to T.k RNAP at high temperatures is not possible (23).

While the thermodynamic impact of increased temperature on enzyme kinetics has been appreciated for many years, the substantial difference in elongation rates at low and high temperatures we observed for nucleotide addition by *T. k*. RNAP was more striking than anticipated (Fig. 3, 5 and 6). In previous characterizations of eukaryotic and bacterial RNAPs assembled on identical scaffolds, we did not observe such rapid formation of intermediate RNA species (3–10). At 25°C, the EC stability, rate constant of elongation, and binding affinity of *T. k*. RNAP disfavor processive transcription elongation. At a temperature permissive for growth, the EC stability coupled with rapid nucleotide addition leads to robust and productive elongation, but at both 25˚C and 65˚C, elongation rates for *T.k*. RNAP are largely insensitive to NTP concentrations.

Our prior characterizations of both single and multiple nucleotide addition events for eukaryotic Pols I, II, and III, as well as mycobacterial RNAPs, all demonstrated some degree of NTP-concentration dependence on the observed rate constants that govern nucleotide addition (4–10). Our finding that *T. k*. RNAP does not demonstrate this otherwise highly conserved enzymatic property of multi-subunit RNAPs, which is perplexing. A meta-analysis of organisms across Eukarya, Bacteria, and Archaea found that all organisms analyzed had intracellular ATP concentrations in the range of 1–10 mM, with the average being ~4 mM (17, 24), suggesting that intracellular concentrations of nucleotides are not significantly different based upon domain of origin. Because of this, we hypothesize that *T. k*. RNAP demonstrates a mechanism of nucleotide addition that is not rate-limited by the available NTPs, but rather is limited by the conformation of the RNAP or potentially by the association of trans-acting factors (absent in our analyses). Our findings highlight the impacts of selective pressures on transcription elongation complex stability and the rate constants governing nucleotide addition at low and high temperatures. The divergence in core catalytic functions of the *T. k*. RNAP from eukaryotic enzymes suggests that novel regulatory strategies may be deployed in the two domains. Different rate-limiting steps will give rise to alternative control mechanisms. Future studies will continue to elucidate the impact of temperature on the molecular mechanisms governing RNA synthesis and the regulatory mechanisms deployed by archaeal RNAPs.

## MATERIALS AND METHODS 

### Buffers

 *In vitro* time courses were performed in reaction Buffer A: (40 mM KCl, 20 mM Tris-Acetate, pH 8.39 at 25°C, 2 mM dithiothreitol (DTT), 0.2 mg·mL^−1^ bovine serum albumin [BSA]); components were prepared from stocks immediately before use. 65°C time courses were performed in the same reaction buffer that was pH adjusted to be 8.39 at 65°C.

### Protein purification

 Purification was performed as previously described (13).

### Oligonucleotides

 All template, non-template, and primer oligonucleotides/oligoribonucleotides were purchased from Integrated DNA Technologies (Coralville, IA). Oligonucleotides were gel-purified, and both DNA and RNA oligonucleotides and oligoribonucleotides were dialyzed against Buffer A (15).

Template DNA sequence:

5′−ACCAGCAGGCCGATTGGGATGGGTATTCCCTCCTGCCTCTCGATGGCTGTAAGTATCCTATAGG−3′

Non-template DNA sequence:

5′−CCTATAGGATACTTACAGCCATCGAGAGGCAGGAGGGAATACCCATCCCAATCGGCCTGCTGGT−3′

RNA primer sequence:

5′-AUCGAGAGG-3′

### Elongation complex stability time courses

Elongation complex stability experiments were performed as previously described (4, 5, 7). Briefly, labeled ECs were mixed with a solution of 750 mM KCl and RNase A. A proportion of intact ECs was normalized to the starting material and plotted without fitting. Indication of the RNase A cleavage site is below:

5′-AUC│GAGAGGC-3′

### Rapid mixing time courses

 Purified *T. k*. RNAP was incubated with pre-annealed RNA:DNA hybrid for 10 minutes at ambient temperature before the addition of the fully complementary non-template DNA and incubated for 10 minutes at ambient temperature to form active ECs. The ECs were then radiolabeled by the addition of α-^32^P-CTP and Mg^2+^ at ambient temperature. The labeling reaction was halted by the addition of EDTA. Radiolabeled ECs were then loaded into the left syringe of the KinTek chemical quench flow (KinTek Corporation, Snow Shoe, PA). Into the right syringe was first a blank of Buffer A for time zero collection, then a solution of ATP or ATP and GTP. The contents of the two syringes were rapidly mixed by the machine in ~2 ms, then the reaction occurred for a user-determined period of time. The reaction is rapidly quenched with 1 M HCl. The quench flow was maintained at either 25°C or 65°C by a constantly circulating water bath set to the given temperature.

 The collected samples were neutralized, then mixed with a formamide storage dye. RNA products were then separated via polyacrylamide gel electrophoresis, imaged, and quantified using phosphorimage analysis (15).

### Data analysis

 At least three time courses for each ATP concentration and ATP and GTP concentrations were used in the final data analysis. The normalized signal intensities of data points were first plotted using KaleidaGraph (Synergy Software, Reading, PA) for model-independent analysis. Single-nucleotide addition time courses collected at 25°C were fit to equation 1 (Fig. 2D).

 Determination of parameter values and calculations of observed rate constants were performed on each data set using the MatLab toolbox MENOTR (Multi-start Evolutionary Nonlinear OpTimizeR). This tool uses a hybrid of non-linear least squares (NLLS) and genetic algorithm analysis to identify the best fit given the data and provided kinetic scheme (6–10, 16).

## SUPPLEMENTAL MATERIAL

Supplemental figures msphere.00319-25-s0001.pdf Control experiments.

ASM does not own the copyrights to Supplemental Material that may be linked to, or accessed through, an article. The authors have granted ASM a non-exclusive, world-wide license to publish the Supplemental Material files. Please contact the corresponding author directly for reuse.
