# Supplementary material for: Kinetic characterization of RNA synthesis catalyzed by the model hyperthermophilic archaeon Thermococcus kodakarensis RNA polymerase
Source: mSphere. 2025 Sep 26;10(10):e00319-25. doi: 10.1128/msphere.00319-25 (PMC12570502; doi:10.1128/msphere.00319-25)
Supplement: Supplemental figures — Control experiments. [file msphere.00319-25-s0001.pdf]

Supplemental Data

A

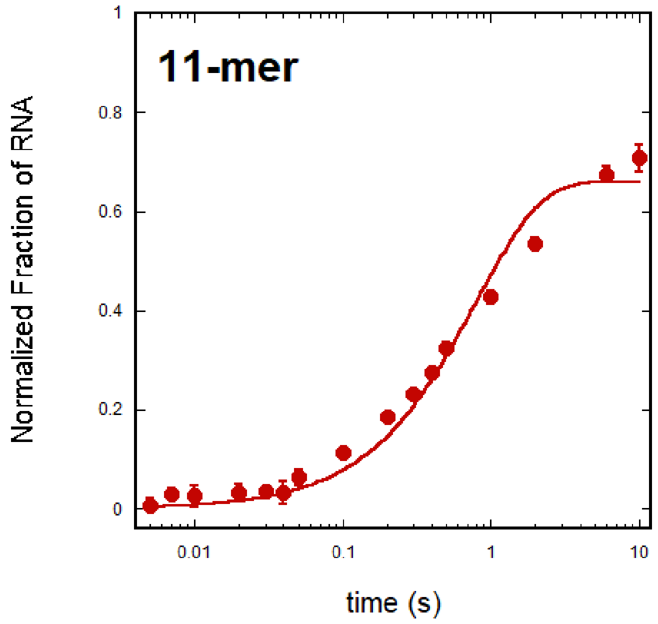

Supplemental Figure S1. Representative plot of 10 μM time course data fit to Eq. 2. Data points are the average of three replicates, error bars represent standard deviation.

A

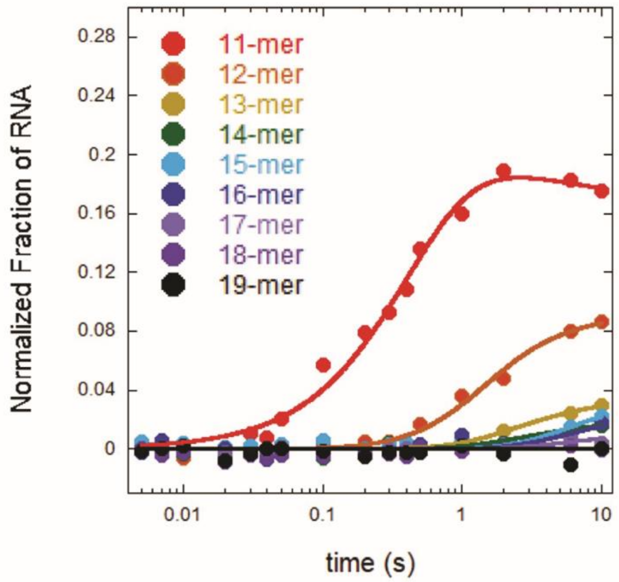

Supplemental Figure S2. Representative plot of all RNA intermediates captured during multiple nucleotide addition events catalyzed by *T. k.* RNAP at 25°C,

highlighting the few detectable intermediate RNAs of length 13-mer+. Data was model-dependently fit using Scheme 2.

A

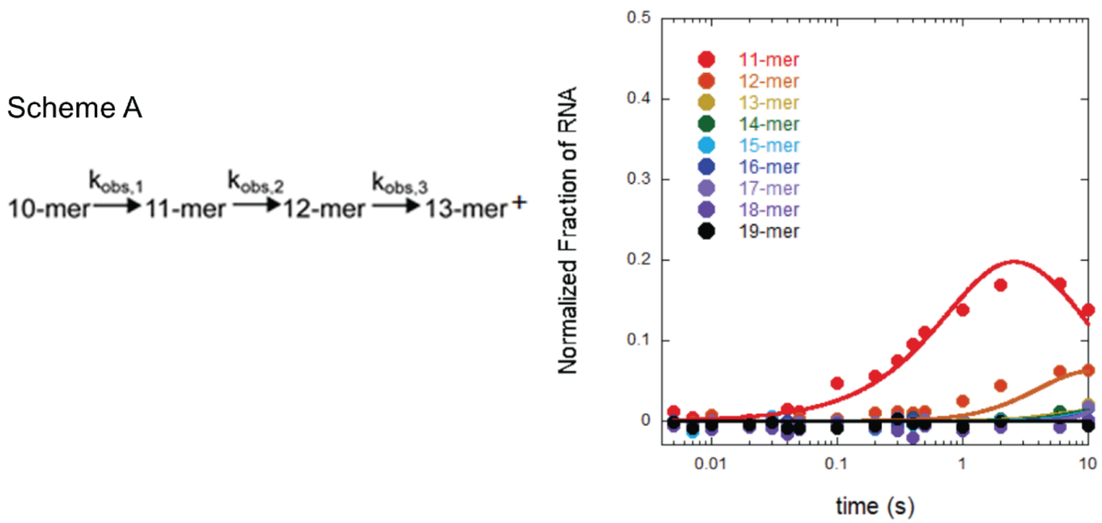

Supplemental Figure S3. Representative plot of RNA intermediates formed at 25°C fit to Scheme A. The fit lines show systematic deviation from the data points, resulting in the use of Scheme 2 for subsequent analyses.
